# Inhibitory Effect of Volatiles Emitted From *Alcaligenes faecalis* N1-4 on *Aspergillus flavus* and Aflatoxins in Storage

**DOI:** 10.3389/fmicb.2019.01419

**Published:** 2019-06-25

**Authors:** An-Dong Gong, Nan-Nan Wu, Xian-Wei Kong, Yi-Mei Zhang, Meng-Jun Hu, Shuang-Jun Gong, Fei-Yan Dong, Jian-Hua Wang, Zhi-Yong Zhao, Yu-Cai Liao

**Affiliations:** ^1^Henan Key Laboratory of Tea Plant Biology, College of Life Sciences, Xinyang Normal University, Xinyang, China; ^2^College of Plant Science and Technology, Huazhong Agricultural University, Wuhan, China; ^3^Department of Plant Science and Landscape Architecture, University of Maryland, College Park, MD, United States; ^4^Institute of Plant Protection and Soil Science, Hubei Academy of Agricultural Sciences, Wuhan, China; ^5^Institute for Agri-Food Standards and Testing Technology, Laboratory of Quality & Safety Risk Assessment for Agro-Products (Shanghai), Ministry of Agriculture, Shanghai Academy of Agricultural Sciences, Shanghai, China

**Keywords:** *Aspergillus flavus*, aflatoxins, *Alcaligenes faecalis*, volatile, dimethyl disulfide, methyl isovalerate

## Abstract

Controlling aflatoxigenic *Aspergillus flavus* and aflatoxins (AFs) in grains and food during storage is a great challenge to humans worldwide. *Alcaligenes faecalis* N1-4 isolated from tea rhizosphere soil can produce abundant antifungal volatiles, and greatly inhibited the growth of *A. flavus* in un-contacted face-to-face dual culture testing. Gas chromatography tandem mass spectrometry revealed that dimethyl disulfide (DMDS) and methyl isovalerate (MI) were two abundant compounds in the volatile profiles of N1-4. DMDS was found to have the highest relative abundance (69.90%, to the total peak area) in N1-4, which prevented the conidia germination and mycelial growth of *A. flavus* at 50 and 100 μL/L, respectively. The effective concentration for MI against *A. flavus* is 200 μL/L. Additionally, Real-time quantitative PCR analysis proved that the expression of 12 important genes in aflatoxin biosynthesis pathway was reduced by these volatiles, and eight genes were down regulated by 4.39 to 32.25-folds compared to control treatment with significant differences. And the *A. flavus* infection and AFs contamination in groundnut, maize, rice and soybean of high water activity were completely inhibited by volatiles from N1-4 in storage. Scanning electron microscope further proved that *A. flavus* conidia inoculated on peanuts surface were severely damaged by volatiles from N1-4. Furthermore, strain N1-4 showed broad and antifungal activity to other six important plant pathogens including *Fusarium graminearum, F. equiseti, Alternaria alternata, Botrytis cinerea, Aspergillus niger*, and *Colletotrichum graminicola.* Thus, *A. faecalis* N1-4 and volatile DMDS and MI may have potential to be used as biocontrol agents to control *A. flavus* and AFs during storage.

## Introduction

*Aspergillus flavus* is an important agricultural fungus which can infect many grain and oil crops in pre- and post-harvest such as groundnuts, maize, rice and cottonseed et al. (Yu et al., 2004). Moreover, some aflatoxigenic *A. flavus* can produce large amount of toxic aflatoxins (AFs) in crops and food, and seriously affect human and livestock health (Waliyar et al., 2015). Currently, 18 types of AFs produced by *Aspergillus* spp. have been identified. Among them, aflatoxin B_1_ (AFB_1_) was considered as the most dangerous mycotoxins to mammals due to its high toxicity and carcinogenicity (Asters et al., 2014; Adhikari et al., 2016). International Agency for Research on Cancer (IARC) classified AFB_1_ as Group I carcinogen to humans (Robens and Cardwell, 2003; Zhuang et al., 2016).

*Aspergillus flavus* and AFs pose great risk of human health worldwide. Williams et al. (2004) estimated that more than 5 billion people are suffering from the chronic exposure to AFs. Vardon calculated that the annual economic losses caused by AFs was about $500 million in the United States (Wu, 2015; Outlaw et al., 2016), and the additional cost of disease management is more than $20–$50 million (Robens and Cardwell, 2003). In less developed countries, because of the poor income and scanty food supplication, AFs contamination in food is even more severe, resulting in serious harms to local people. In Burundi and Congo, the percentage of AFs contamination was 100% in 244 samples collected from local marker including crops, milk, and other products. In these samples, more than 50% crops were contaminated with large amount of AFs which were higher than the maximum limit set by the European Union (Udomkun et al., 2018). Azziz-Baumgartner et al. (2005) reported over 125 human deaths in Kenya due to the consumption of AFs contaminated foods during 2004–2005. More seriously, in hepatitis B/C virus endemic countries (such as China, India, Africa, etc.), AFs led to severe hazards to humans. Taking China as an example, under the dual pressure of hepatitis B/C virus and AFs, more than 3.7 million people died of liver cancer every year. This accounts for 50% of the world’s liver cancer deaths (Chen et al., 2013).

Because of the great damage and serious risks to humans, many countries have legislatively defined the maximum levels of AFs intended for human and animal consumption. For example, in groundnut and their products, United States Food and Drug Administration permits the maximum AFs at 20 ppb (Torres et al., 2014). In European Union, maximum level is 4 ppb for AFs, and 2 ppb for AFB_1_ (European Commission [EC], 2010). Upper limit of AFs is 10 and 15 ppb in Kenya (Wagacha et al., 2013) and Malaysia (Leong et al., 2010), respectively. In China, the maximum level for AFB_1_ is 20 ppb, and total AFs has not yet regulated. No regulation for total AFs and AFB_1_ in some developing counties (Kamika and Takoy, 2011).

Although legislations aimed to reduce the potential damage of AFs to humans is necessary, farmers in poor areas can not afford to monitor AFs (Probst et al., 2010). Thus, the effective strategies to eliminate AFs is controlling the initial infection of *A. flavus* in crops and grains (Abdel-Kareem et al., 2019). The *A. flavus* control in storage currently relies on physical drying, infected kernel sorting and chemical agents (Nurtjahtja, 2019). While the first two methods are safe and easy strategies and have been used for a long period (Bediako et al., 2018), the synthetic chemical agents may cause adverse effects on human health and environment due to long degradation period, toxic residues and potential undesirable biological effects (Prakash et al., 2015; Li et al., 2016). Bio-active compounds from organisms have been gradually applied in controlling *A. flavus* and AFs during storage (Kai et al., 2009; Passone and Etcheverry, 2014). Some essential oils extracted from plants have been widely used as promising agents in controlling *A. flavus* and AFs during storage, such as *Curcuma longa* (Hu et al., 2017), *Litsea cubeba* (Li et al., 2016), *Zanthoxylum molle* (Tian et al., 2014), *Cinnamomum jensenianum* (Tian et al., 2012), *Peumus boldus, Lippia turbinata* (Passone and Etcheverry, 2014), *Oxalis corniculata* (Rehman et al., 2015) and *Cuminum cyminum* (Kedia et al., 2014). Some compounds have been produced into commercial agents (DMC base Natural, owned by DOMCA S.A.), and successfully applied in disease control.

Despite the potential of plant extracts (i.e., bio-compounds and oils), plant growth requires a long period, dedicated maintenance and large cultivated areas. Moreover, extraction of bio-active compounds needs a large amount of plant sources which are not always available. Compared to plant materials, microbes are simple to obtain and culture, which are easier to obtain large amount. Till now, several microbes have been obtained and used in controlling *A. flavus* and AFs such as *Bacillus megaterium* (Chen et al., 2019), *B. pumilus* (Cho et al., 2009) and non-aflatoxigenic *A. flavus* strains (Ehrlich, 2013; Zanon et al., 2016). However, the biocontrol activity of these microbes mainly relies on antagonism (macromolecular metabolite) and competition, which always required direct contact with grains. Such contact may lead to secondary pollution to grains in storage. Compared to these contact phase, volatile phase can easily and uniformly distribute in the whole space of storage, and generated lower residual after volatilization. Recently, some evidences proved that the inhibitory effect of volatile phase on *A. flavus* in storage was consistently more effective than contact phase (Hu et al., 2017; Ma et al., 2017). To date, seldom microbe volatile was identified and used to control *A. flavus* and AFs during storage. Thus, the objectives of this study were to: (1) screening effective biocontrol microbes against *A. flavus* through the production of volatiles; (2) evaluating biocontrol efficacy of microbes against *A. flavus* and AFs in grain and food during storage; (3) identifying the primary antifungal bio-active volatiles; and (4) analyzing their antagonistic effect on cell ultra-structure of *A. flavus* and AFs biosynthesis.

## Materials and Methods

### Microorganism and Plant Materials

*Alcaligenes faecalis* N1-4 was isolated from rhizosphere soil of tea plant in Cheyun mountain, Xinyang city, Henan, China (Fang et al., 2014). The bacteria was suspended in 25% (v/v) glycerol and stored at -80°C for long-term use.

Regular-sized maize (cultivar Zhengdan 958), peanut (Silihong) and soybean (W82) were purchased from local markets. Rice (Huanghuazhan) samples were supported by Huazhong Agricultural University. These samples were divided into two parts, and adjusted water activity (a_w_) to 0.8 and 0.9, respectively. Then, they were inoculated with *A. flavus* to detect the disease development and AFs contamination in storage.

Seven important phyto-pathogens were used in our test. These fungal strains included *A. flavus, Fusarium graminearum, F. equiseti, Alternaria alternata, Botrytis cinerea, Aspergillus niger*, and *Colletotrichum graminicola.* The pathogens were isolated from infected plants and stored in our lab for broad antifungal tests (Gong et al., 2014). *A. flavus* conidia was inoculated into PDB medium and cultured at 28°C for 48 h to produce mycelium coils.

### Screening of Bacteria With Antifungal Volatile Production

The soil samples were collected from tea gardens containing large amounts of active humus. The soil was suspended in sterilized water and diluted to 10^-5^. The suspension (100 μL) was spread on the surface of NA medium and cultured at 28 for 48 h. The obtained microbe clones were streaked on NA medium to produce pure culture. Bacteria bodies on NA surface were washed off and co-cultured with *A. flavus* in Face-to-face (FTF) cultural method to screen antagonistic bacteria.

In FTF tests, *A. flavus* mycelia coil was placed to the center of PDA medium bacteria bodies (OD600 = 1.8, 100 μL) were spread on NA medium. The two petri dishes were placed FTF with *A. flavus* above and bacterial plate at the bottom. PDA plate inoculated with *A. flavus* mycelia challenged with NA plate was used as control. All sets were cultured at darkness and 28°C for 3 days. The mycelium diameter in each group was recorded, and the inhibitory rate was calculated following the forma below.

Inhibitory rate (%) = [(the diameter of control – the diameter of antagonist treatment)/the diameter of control] × 100.

Inhibitory effect of bacteria to *A. flavus* conidia germination was measured. Ten micro liter of *A. flavus* conidia (10^5^ cfu/mL) were injected on a round filter paper (5 mm in diameter), and placed to the center of PDA plate. Isolated bacteria were spread on NA plate. The two petri dishes were located FTF with PDA above. PDA medium inoculated with *A. flavus* conidia was used as control. All sets were cultured at darkness and 28°C for 3 days. The diameter of conidia in each test was recorded, and the inhibition rate was calculated as before.

### DNA Extraction and Phylogenetic Tree Analysis

Single clone of N1-4 was inoculated into NB medium and cultured at 200 rpm and 28°C for 48 h. The cultured broth was centrifuged at 12,000 rpm for 10 min, and the collected cell bodies were used for DNA extraction (Gong et al., 2014). The 16S rRNA sequences of strain N1-4 were amplified using two universal primers (27 f and 1541 r). Obtained 16S rRNA were sequenced in TIANYI HUIYUAN company, and blasted in GenBank database. The strains in GenBank database homologous to N1-4 were selected and used to construct the phylogenetic tree through MEGA program and neighbor-joining method (Ding et al., 2015; Chen et al., 2017).

### Biochemical Analysis of Strain N1-4

Biochemical activity of N1-4 was conducted through BIOLOG MicroStation^TM^ System (Biolog Inc., United States). Exactly, strain N1-4 was streaked on NA medium. Fresh single clone was transferred into IF-A GEN III Inoculating Fluid. Then the suspension was transferred into GEN III MicroPlate with 120 μL in each cell. The plate was cultured at 37°C in darkness for 12 h, and the optical density of each cell in MicroPlate was recorded in BIOLOG MicroStation^TM^. All recorded data were aligned in bacterial database through BIOLOG MicroStation^TM^ System (Banik et al., 2016).

### Identification of Volatiles From N1-4

Volatiles from N1-4 were enriched with solid-phase micro-extraction (SPME) fiber (divinylbenzene/carboxen/ polydimethlsiloxane), and identified through GC-MS/MS system (5975B-7890N, Agilent Technologies Inc.). N1-4 cell were spread on the surface of NA medium in a 150 mL flask. The flask containing NA medium without N1-4 bodies was used as control. Each flask was sealed with two layer of membrane, and placed at 37°C, and darkness for 24 h to produce volatiles. Volatiles in airspace of each flask were enriched with SPME system for 30 min, then analyzed with GC-MS/MS system equipped with an Agilent HP-5MS fused-C18 capillary column (length 30 m, internal diameter 0.25 mm, 0.25 μm thickness film). The samples were analyzed with splitless injection mode. The inlet temperature was 250°C, carrier gas was helium. The compounds were analyzed using the method as follows: initial temperature at 40°C for 3 min, following a linear heating rate of 3°C ⋅ min^-1^ to 160°C, and contained for 2 min, 8°C ⋅ min^-1^ to 220°C, and contained for 3 min. Each sample was conducted for two times. The compounds in N1-4 samples that were not present in the control samples were considered to be the final analyzes.

### Minimal Inhibitory Concentration Analysis of Identified Volatiles

Identified compounds in N1-4 volatiles were selected for MIC analysis against *A. flavus* in FTF test (Gong et al., 2015). Ten micro liter fresh *A. flavus* conidia (5 × 10^5^ cfu/mL) were inoculated to a round paper disk placed in the center of PDA plate. Another plate was inoculated with identified compound, respectively. Ethanol was used as control. All compounds was adjusted to the final concentration of 5, 10, 100 and 200 μg/L (compound weight to airspace volume), respectively. These two dishes were placed FTF and incubated at 28°C and darkness for 4 days. The diameter of *A. flavus* mycelium was calculated 4 dpi, and the inhibitory rate was calculated according to the following formula: Inhibitory rate (%) = [(the mycelia diameter of control – the diameter of N1-4 treatment)/the diameter of control] × 100.

### Infection Control and AFs Prevention by N1-4 in Crops During Storage

Peanuts, maize, rice and soybean kernels were inoculated with *A. flavus* conidia, and challenged with N1-4 in sealed FTF culture test to detect the inhibitory effect of volatiles. In the tests, eight flasks was prepared, and two flasks as a group for one kind of kernel. 100 g of grains was added into each flask and autoclaved at 121°C and 1.01 MPa for 20 min, and cooled down to room temperature. 100 μL *A. flavus* conidia (at 1 × 10^5^ cfu/mL) was added to each flask and mixed well. The two flasks in each group was inoculated with 10 mL (5 mL) of sterilized water, mixed well, and adjusted a_w_ to 0.9 (0.8) with Aqualab Series 3 model TE (Decagon Devices, Pullman, WA, United States), respectively.

Peanuts, maize, rice and soybean kernels in each flask were equally divided into two parts, and placed into two petri dishes, respectively. One petri dish was challenged FTF with strain N1-4 coated on NA medium. The other petri dish challenged with NA medium was used as control. All dishes were cultured at 28°C and darkness for 7 days. The disease incidence of each treatment was recorded. The grains were collected, dried at 60°C for 4 days, and milled well for AFs extraction.

### Quantitative Analysis of AFs

Milled samples of each grain were used for AFs extraction and quantitative analysis. One gram of milled sample was re-suspended in 5 ml acetonitrile/water (84/16, v/v), and vortex for 5 min. The suspension was centrifuged at 12,000 rpm for 10 min. The supernatant was transferred into a new tube, and same volume of hexane was added into the tube, mixed well. The upper layer was obtained and used for AFs analysis. The quantitative analysis of AFs were conducted through LC-ESI-MS system containing Thermo Surveyor plus HPLC system coupled to a TSQ Quantum Ultra mass spectrometer (Thermo Scientific, CA, United States) (Warth et al., 2012). AFs (AFB_1_, AFB_2_, AFG_1_, and AFG_2_) purchased from Sigma (Sigma-Aldrich, St. Louis, MO, United States) were used as standards for quantitative analysis.

### Ultra-Structural Analysis of *A. flavus* Cell on Peanuts Surface

Ultra-structural of *A. flavus* cell on peanuts coat of a_w_ 0.92 were analyzed through JSM-4800 scanning electron microscope (SEM, Hitachi, Tokyo, Japan). Peanuts in control and N1-4 treatments were fixed with 0.1% (v/v) osmic acid for 1 h. A piece (3∼5 mm × 3∼5 mm) of peanut coat was ripped down, and affixed to SEM stubs, respectively. Then, all samples were sprayed with gold and examined in SEM (Boukaew and Prasertsan, 2014).

### Broad Spectrum Inhibitory Activity of Strain N1-4

Broad antifungal tests of N1-4 against other 6 fungal pathogens were conducted with FTF method as mentioned above. Fresh hyphae block (5 cm in diameter) was inoculated to the center of PDA medium, respectively. The plate was challenged with strain N1-4 on NA plate with FTF method, respectively. All tests were cultured at 28°C and darkness for 5 days. The inhibition rate was calculated according the methods above.

### RNA Extraction and Quantitative Real-Time PCR Analysis

Fresh *A. flavus* conidia (100 μl, 10^6^ cfu/mL) were spread on PDA medium in one petri dish. Another dish containing NA medium was spread with N1-4 cell bodies (10^8^ cfu/mL, 100 μl). Then, the two dishes were challenged FTF with *A. flavus* on the top. *A. flavus* conidia on PDA plate challenged with NA medium was used as control. All treatments were cultured at 28°C and darkness for 48 h. The mycelia on PDA surface were collect and used for RNA extraction.

Total RNA in *A. flavus* mycelia was extracted by Trizol (Invitrogen) method (Li et al., 2017). The RNA was treated with DNase at a concentration of 1.5 unit/μg. cDNA was synthesized using a PrimeScript^TM^ RT Reagent Kit (Takara). The cDNA products were diluted 20-fold with nuclease free deionized water. Reverse transcription quantitative PCR (RT-qPCR) was performed using a Bio-Rad iQ2 PCR system (Bio-Rad, United States). The PCR conditions were as follows: 95°C for 1 min; 40 cycles of 95°C for 20 s, 60°C for 20 s, and 72°C for 30 s. Twelve important genes in AFs biosynthesis pathway were used in our tests including *aflR, AccC, aflCa, aflA, aflS, aflO, aflD, aflF, aflP, aflQ, aflX*, and *aflC* (Cleveland et al., 2009; Hua et al., 2014). β*-tubulin* was used as the endogenous control due to its relatively stable expression level. All primers used for RT-qPCR were shown in Table 1. The relative expression of genes in N1-4 compared to control was calculated by using the 2^∧^-ΔΔCt method (Chen et al., 2017; Chai et al., 2018).

**Table 1:** Primers used in RT-qPCR analysis.

Gene name	Gene functions	Primers used in the RT-qPCR
*aflR*	Pathway regulator	F: AGCACTACAAACACTGACCCAC R: CCAGCACCTTGAGAACGATAA
*aflCa*	Noranthrone monooxygenase, Norsolorinate-anthrone to norsolorinate	F: GCACCAATGGAGCCGTAT R: GCGGTGTTCGTAGCGTTC
*aflA*	FAS alpha subunit, Acetate to polyketide	F: CGTGAGGTCAAGGCATTCCT R: GACTTGGCCCCCCTTCTGT
*aflS*	Transcription enhancer, Pathway regulator	F: CCGAAGATTCCGCTTGGA R: TGAAGACATGCAGCAAAAGGA
*aflO*	O-methyltransferase B, dihydrodemethylsterigmatocystin to dihydrosterigmatocystin	F: TGCTGTGGCATCCATTCAAA R: GGACTGCGTCTTCCAAAAGG
*aflD*	NOR reductase, norsolorinic acid to averantin	ACTGCGACTCGGAAACTGATG TGCTCCTCCCGCAATGTC
*aflP*	O-methyltransferase A, sterigmatocystin to O-methylsterigmatocystin	F: TGTGTCGAGTGATGTGGGACTAG R: GCCACCCAGCTCAACCTACA
*aflF*	NOR dehydrogenase, norsolorinic acid to averantin	F: AAGATGCTGGGCACGTTTG R: CATGGGTGAGGACGAATTGG
*aflQ*	Oxydoreductase, O-methylsterigmatocystin to AFB_1_ and AFG_1_, dihydro-Omethylsterigmatocystin to AFB_2_ and AFG_2_	F: TTGCTGGGCTTGTGGATTC R: GAGGAGGACGCGTGTCTTTG
*aflC*	polyketide synthases, Acetate to polyketide	F: TCACAAGCGATGCACAGTTG R: AACTGACGAATGTGGGTCTTGTACT
*aflX*	Monooxygenase/oxidase, VA to DMST	F: ACCGCGTTGCACATCGT R: TGGGTGTCCACAACCTTCGT
*AccC*	Acetyl-CoA carbosylase, Acetyl-CoA to Malonlyl-CoA	F: ATGGTAAGACCTGCCTGCTA R: AGCGAGGATACCGAGGAT
β*-tubulin*	Endogenous control, Reference gene	F: AGCAGGCGAAGAAGGAGG R: ACGCCACGCATTTGATCTTC

### Data Analysis

All statistical analyses were performed by one-way analysis of variance (ANOVA) with SPSS 17.0 statistics software for windows (SPSS Inc., Chicago, IL, United States). Experiments were arranged in a completely randomized design with at least two replications. Mean comparisons were performed with Student’s *t* test at *P* < 0.05.

## Results

### Isolation of Active Bacteria With Antifungal Volatile Production

In screening tests, 693 bacteria were isolated from tea soil, and six bacteria showed great antifungal activity against *A. flavus* in FTF tests. Among these strains, N1-4 could completely inhibit the growth of *A. flavus* mycelium, as well as conidia germination *in vitro*. N1-4 was selected for further biocontrol analysis in the tests.

**FIGURE 1:**
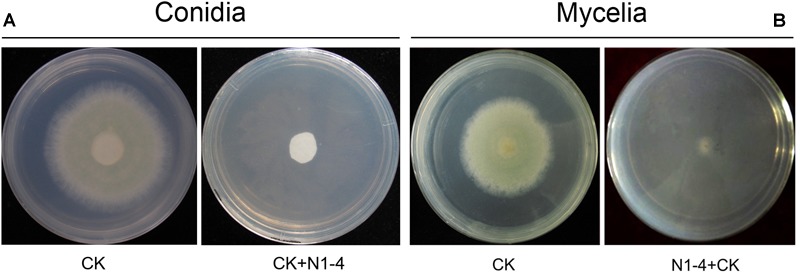
Antifungal activity of strain N1-4 against the growth of *A. flavus* mycelia and conidia. Mycelia pellet and conidia of *A. flavus* was inoculated to the center of PDA plate was used as control (CK), respectively. *A. flavus* challenged with bacteria N1-4 (CK+N1-4) with FTF method was used to test the antifungal activity, respectively. **(A)** The inhibitory effect of N1-4 against conidia germination of *A. flavus*. **(B)** The inhibitory effect of N1-4 against mycelia growth of *A. flavus*.

In control treatment, mycelia in PDA medium could grow quickly, and extended to 5.2 cm in diameter 5 days post inoculation (dpi). Conidia on PDA surface germinated to hyphae, and extended quickly. When challenged with bacteria, N1-4 in NA medium without contact to *A. flavus* cell, can greatly inhibited the conidia germination, as well as mycelia growth of *A. flavus* (Figure 1). Seldom fresh mycelia were formed in N1-4 treatment 5 dpi. Hence, the inhibition rate of N1-4 to mycelia growth and conidia germination was up to 100% compared to control (Figure 1).

**FIGURE 2:**
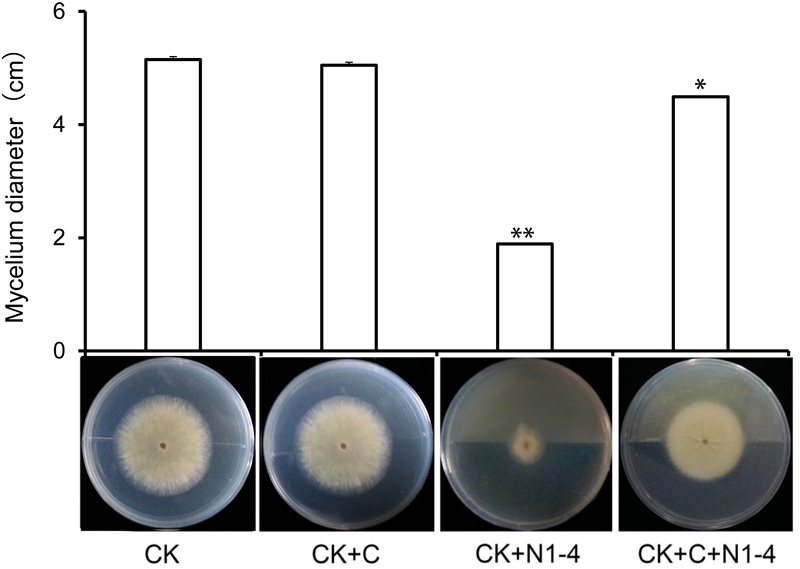
Antifungal activity of volatiles from strain N1-4 against *A. flavus*. Mycelia pellet of *A. flavus* was inoculated to the center of PDA plate was used as control (CK). *A. flavus* challenged with active charcoal (CK+C), N1-4 (CK+N1-4), and both of them (CK+C+N1-4), respectively. ^∗^ and ^∗∗^ mean significant difference at *p* < 0.05 and *p* < 0.01 leveles compared to CK treatment, respectively.

When active charcoal added into the tests, the diameter of *A. flavus* mycelia is relative equal to control treatment without the presence of N1-4. It means that active charcoal showed no inhibitory effect to *A. flavus*. When N1-4 added into the reaction, the diameter of *A. flavus* (CK+C+N1-4) is shorter than charcoal treatment (CK+C), but longer than N1-4 (CK+N1-4) (Figure 2). Thus, we could deduce that strain N1-4 can produce abundant volatiles, spread in the whole cultural space, and eventually inhibited the growth of *A. flavus*.

### Taxonomic Identification of Bio-Active Strain N1-4

**FIGURE 3:**
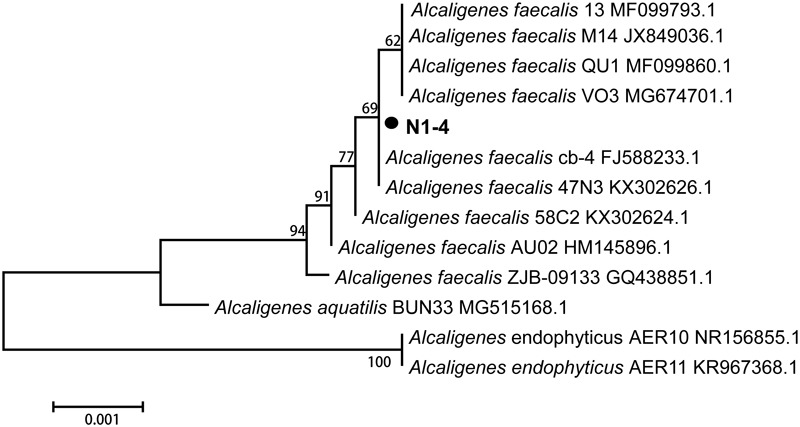
Phylogenetic tree analysis of N1-4 and other homologous strains based on 16S rRNA sequences. The tree was constructed with neighbor-joining methods and the scale bar represents the number of substitutions per base position.

The 16S rRNA sequence of strain N1-4 was sequenced and blasted in GenBank database. The results indicated that sequences of N1-4 showed great similarity to the species of *Alcaligenes faecalis, A. aquatilis* and *A. endophyticus.* The physiological tree was constructed based on 16S rRNA from N1-4 and other homologous strains (Figure 3). Strain N1-4 was classified into a sub-cluster with *A. faecalis*, and showed highly homologous and close genetic distance to *A. faecalis* cb-4 (FJ588233.1) and *A. faecalis* 47N3 (KX302626.1). Thus, we deduced that strain N1-4 was initially identified to be *A. faecalis* based on 16S rRNA sequences.

Biochemical analysis further proved that strain N1-4 exhibited great similarity to *A. faecalis* strain in BIOLOG MicroStation^TM^ System. Both of them can utilize more than 18 kinds of nutrients including methyl pyruvate, L-alanine, L-lactic acid and L-aspartic acid so on. They could grow at the presence of 1∼4% NaCl, and tolerant to 14 kinds of antibiotic such as troleandomycin, lincomycin, vancomycin, and rifamycin SV so on. But, bacteria N1-4 showed different activity to *A. faecalis* on the application of Tween 40 and D-Arabitol, as well as tolerance to 8% NaCl (Table 2).

**Table 2:** Biochemical analysis of strain N1-4 through BIOLOG MicroStation^TM^ System.

Reaction	N1-4	*A. faecalis*	Reaction	N1-4	*A. faecalis*
**Carbon utilization**			**NaCl tolerance**		
p-HydroxyPhenylacetic Acid	++	++	1% NaCl	++	++
Tween 40	-	+	4% NaCl	+	++
Methyl Pyruvate	++	+	8% NaCl	-	+
D-Arabitol	+	-	**PH tolerance**		
L-Alanine	++	+	pH 6	++	++
L-Lactic Acid	++	+	**Compounds sensitivity**		
β-Hydroxy-D,L Butyric Acid	++	+	Troleandomycin	+	++
L-Aspartic Acid	++	+	Lincomycin	++	++
Citric Acid	++	++	Vancomycin	++	++
α-Ketobutyric Acid	+	+	Rifamycin SV	++	++
L-Glutamic Acid	++	++	Minocycline	+	++
α-Ketoglutaric Acid	++	+	1% Sodium Lactate	++	++
L-Histidine	++	++	Aztreonam	+	+
Propionic Acid	++	+	Fusidic Acid	++	++
L-Pyroglutamic Acid	++	+	Guanidine HCl	+	++
L-Malic Acid	++	++	Tetrazolium Viole	++	++
Acetic Acid	++	++	Lithium Chloride	++	++
D-Serine	+	+	D-Serine	++	++
Bromosuccinic Acid	++	+	Niaproof 4	++	++
Formic Acid	+	+	Tetrazolium Blue	++	++

*Alcaligenes faecalis with the greatest similarity to N1-4 was selected as positive species from BIOLOG MicroStation^*TM*^ database. + means positive reaction, ++ means strong positive reaction, - means negative reactions.*

### Identification of Antifungal Volatiles From N1-4

**FIGURE 4:**
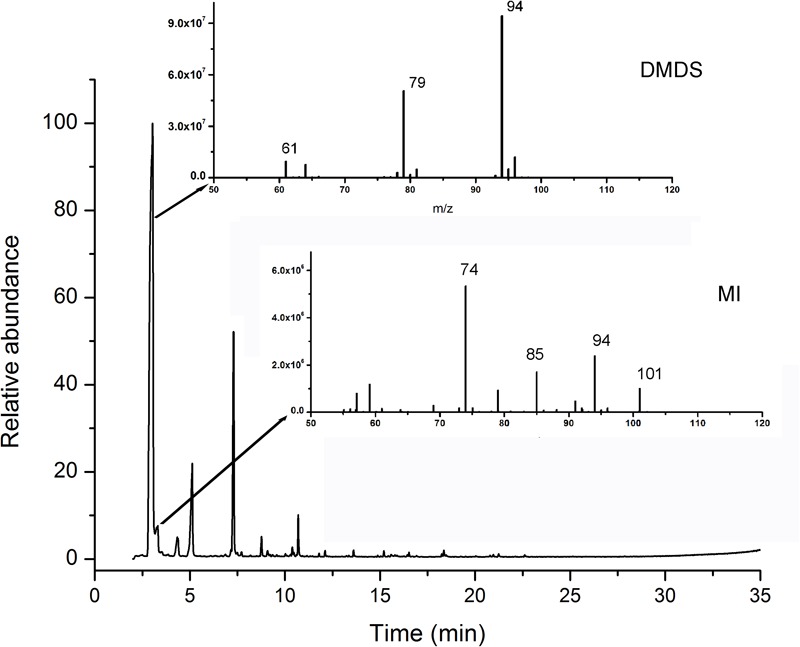
Identification of volatile compounds from strain N1-4 through GC-MS/MS. Volatiles were enriched with SPME system, and identified through GC-MS/MS system. Two compounds, DMDS and MI, in N1-4 profiles and without presence in NA profiles were considered as authentic compounds.

More than 25 compounds were detected in the volatiles of N1-4 through SPME coupled with GC-MS/MS system. But, 23 fractions were also detected in control treatment. Only two compounds, including disulfide dimethyl (DMDS) and methyl isovalerate (MI) were proved exclusive in N1-4 volatiles. In the chromatogram, the retention time of DMDS and MI is between 3.04 and 4.34 min. And, both of them contain low molecular weight between 94 and 130 Dalton. DMDS showed the greatest relative abundance (compared to the total peak area) at 69.90%, which was higher than MI (3.38%) (Figure 4). Additionally, we purchased these two standards for further antagonistic effect analysis.

### MIC Analysis of Identified Volatile Compounds

**FIGURE 5:**
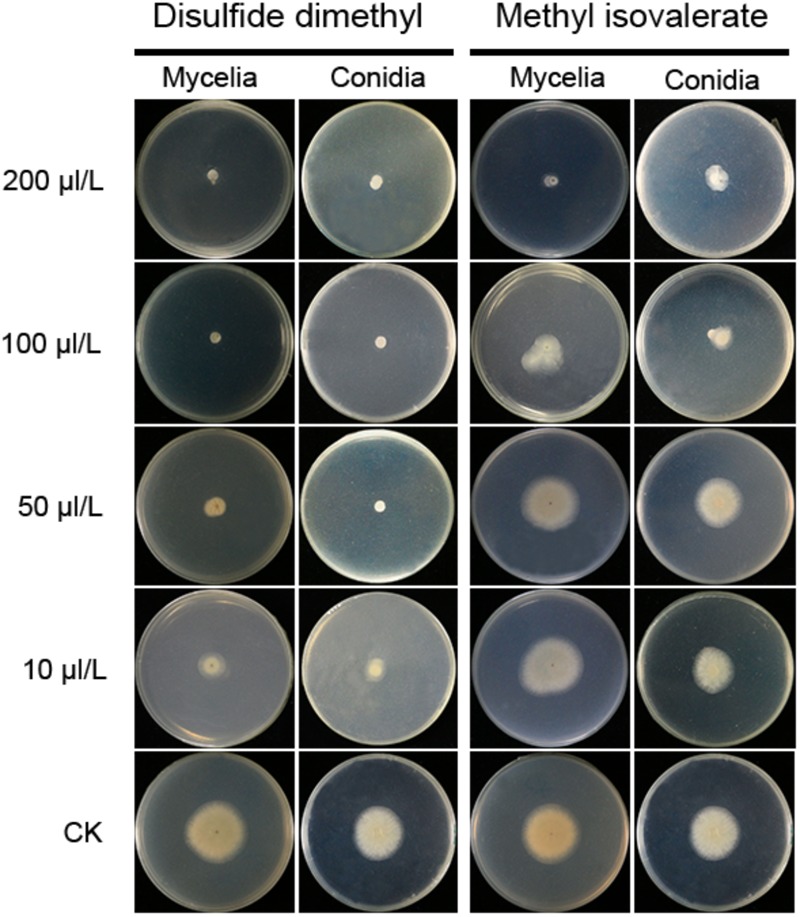
Minimal inhibitory concentration analysis of identified volatiles against mycelia growth and conidia germination of *A. flavus*. Two individual compounds DMDS and MI were diluted to gradient concentrations, and co-cultured with *A. flavus* conidia and mycelia with FTF methods.

Two compounds, DMDS and MI, were serially diluted to 10, 50, 100, and 200 μL/L, and tested their inhibitory effect against *A. flavus* in FTF dual culture tests. In control treatment, *A. flavus* conidia germinated to hyphae, and mycelia grew quickly without the presence of volatiles. When DMDS or MI added into the treatment, the growth of *A. flavus* was greatly inhibited. Obviously, at 50 μL/L or higher concentration, DMDS can eventually prevent conidia germination of *A. flavus*. And at 100 μL/L or higher concentration, DMDS eventually prevented the growth of mycelia. Thus, the MIC of DMDS against conidia germination and mycelia growth of *A. flavus* was 50 and 100 μL/L, respectively. The MIC of MI against mycelia growth of *A. flavus* was 200 μL/L, but can not eventually prevent the germination of conidia (Figure 5). Thus, we can conclude that DMDS with the highest relative abundance (69.90%) and best antifungal activity is the main inhibitory factor in N1-4 volatiles.

### Bio-Control Activity of N1-4 Against *A. flavus* and AFs in Grains During Storage

**FIGURE 6:**
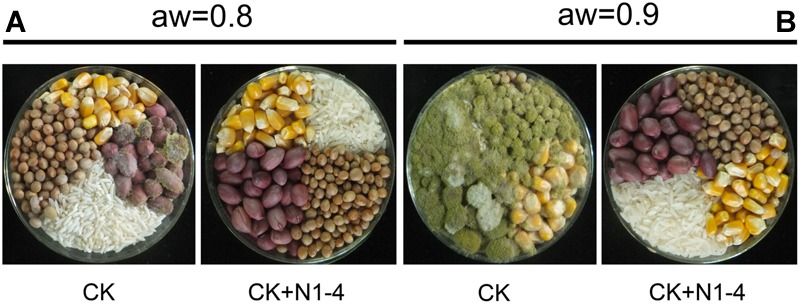
Biocontrol efficacy of volatiles from N1-4 against *A. flavus* and AFs on four different grains of a_w_ 0.8 to 0.9. **(A)** Disease symptom of *A. flavus* inoculated grains with (N1-4+CK) or without (CK) the presence of strain N1-4 at 28°C for 7 days. **(B)** AFB_1_ and AFB_2_ in peanut samples were detected using LC-ESI-MS system.

Volatiles from N1-4 can eventually control *A. flavus* infection in maize, peanut, soybean and rice grains during storage. In control treatment, the *A. flavus* conidia germinated to hyphae, and quickly infected these grains at high a_w_. And the disease severity in peanuts was more serious than that in maize, soybean and rice samples. Additionally, in each grain, the disease severity of *A. flavus* at a_w_ 0.9 was more severe than that at a_w_ 0.8. Take peanuts as an example, the disease incidence in peanuts of a_w_ 0.9 is up to 100% (Figure 6). And a mass of fresh conidia were formed and covered the grain surface. But, in N1-4 added treatment, *A. flavus* conidia did not germinate and infect these grains, and no disease symptom was shown in each grains of two a_w_. Hence, we could deduce that volatiles from N1-4 can greatly inhibit infection development in different grains during storage.

Aflatoxins concentration in four grains showed similar tendency to infected symptom of *A. flavus*. In control treatment of a_w_ 0.9, the total AFs in peanuts is 45.51 μg/g (AFB_1_ is 42.63 μg/g), the concentration is higher than that other grains including maize 37.54 μg/g (AFB_1_ is 34.80 μg/g), rice 22.05 μg/g (AFB_1_ is 20.86 μg/g) and soybean 2.13 μg/g (AFB_1_ is 2.01 μg/g). In control of a_w_ 0.8, less AFs was detected in peanuts (0.37 μg/g), rice (0.01 μg/g) and soybean (0.01 μg/g), respectively. More importantly, no AFs was detected in each grain of 0.8 and a_w_ 0.9 with the presence of N1-4. These results indicate that volatiles from N1-4 not only inhibit the infection of *A. flavus*, it also eventually prevented the contamination of AFs in storage (Table 3).

**Table 3:** Quantitative analysis of aflatoxins (AFs) in infected grains with or without the challenge of N1-4.

a_w_	Treatment	Samples	AFB_1_	AFB_2_	Total AFs
0.8–0.85	CK	peanut	0.37 ± 0.00^∗^	0.00	0.37
		maize	0.00	0.00	0.00
		rice	0.01 ± 0.00	0.00	0.01
		soybean	0.01 ± 0.00	0.00	0.01
	N1-4	peanut	0.00	0.00	0.00
		maize	0.00	0.00	0.00
		rice	0.00	0.00	0.00
		soybean	0.00	0.00	0.00
0.9–0.94	CK	peanut	42.63 ± 1.13^∗^	2.88 ± 0.01^∗^	45.51
		maize	34.80 ± 0.27^∗^	2.74 ± 0.08^∗^	37.54
		rice	20.86 ± 0.01^∗^	1.19 ± 0.03^∗^	22.05
		soybean	2.01 ± 0.04^∗^	0.12 ± 0.01^∗^	2.13
	N1-4	peanut	0.00	0.00	0.00
		maize	0.00	0.00	0.00
		rice	0.00	0.00	0.00
		soybean	0.00	0.00	0.00

*Four grains including maize, peanut, soybean and rice were adjusted to proper a_*w*_, inoculated with *A. flavus* conidia and co-cultured with N1-4 in sealed airspace. Asterisk indicated statistical significance at *P* < 0.05.*

### Structural Analysis of *A. flavus* Inoculated on Peanut Surface

**FIGURE 7:**
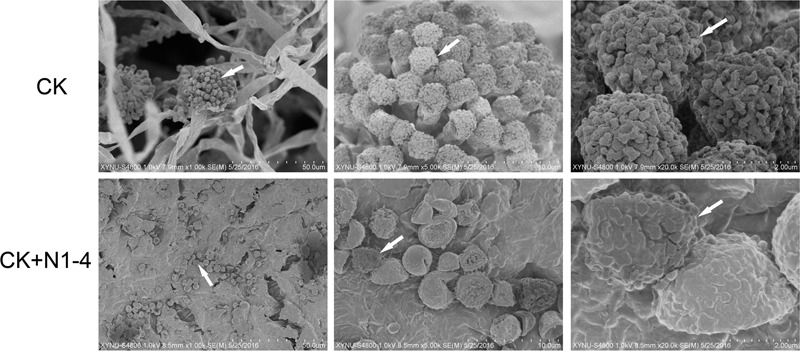
Scanning electron microscope analysis of *A. flavus* cell structure on peanut surface. Peanuts of a_w_ 0.92 inoculated with *A. flavus* conidia was challenged with (N1-4+CK) or without (CK) the presence of strain N1-4 in FTF tests 7 dpi. Co, conidia; cp, conidiophore; my, mycelia; sg, sterigma.

In scanning electron microscopy (SEM) analysis, *A. flavus* conidia germinated to hyphae, formed typical conidiophores, and produce abundant fresh conidia in control treatment (Figure 7). The hyphae and produced conidia covered the whole surface of inoculated peanut coat. But, the conidia in N1-4 added treatment, few conidia were found on peanut coat surface. These conidia were originated from the initial inoculation test. The germination of these conidia were completely inhibited by N1-4, they could not infect grains, produce conidiophore and fresh conidiospore, as well as induce secondary infection on grains. Additionally, these conidia appeared atypical structure with irregular surface and curving bodies. The regular ball around conidia surface were sunk and severely deformed. Finally, we deduce that strain N1-4 with effective antifungal volatiles production endowed valid functions in control *A. flavus* and AFs in grains during storage.

### Broad Spectrum Antifungal Activity of Strain N1-4

**FIGURE 8:**
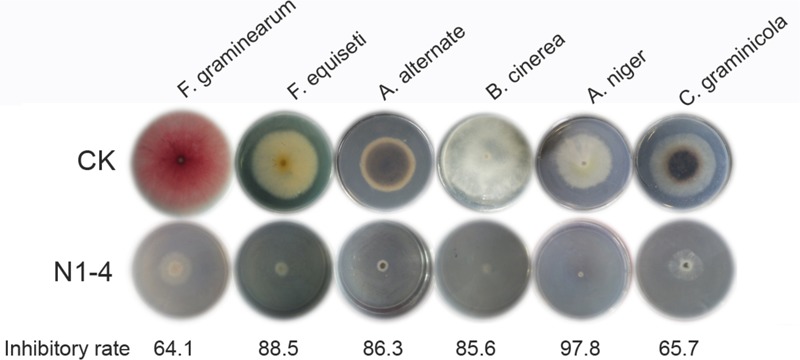
Broad spectrum antifungal activity of strain N1-4 against six important fungal pathogens. Fresh fungal block were inoculated to the center of PDA plate and challenged with strain N1-4 in FTF test, respectively. The diameter of each fungus on PDA plate was measured to calculate the inhibition rate.

Strain N1-4 was co-cultured with other six fungal pathogens by FTF methods to detect the broad antifungal activity. These fungi caused great damage to plant in pre- or post-harvest including *Fusarium graminearum, F. equiseti, Alternaria alternata, Botrytis cinerea, Aspergillus niger*, and *Colletotrichum graminicola.* The inhibitory rate was calculated 5 dpi. As shown in Figure 8, the mycelia in control grew quickly, and extended to 7 cm in length 5 dpi. And some pathogens including *F. graminearum, A. alternata* and *C. graminicola* produced different color of pigments in PDA medium, respectively. When N1-4 was added, the growth of all selected fungi was greatly inhibited by the produced volatiles. The inhibitory rate was ranged between 64.1 and 97.8%, respectively. These results proved that volatiles emitted from strain N1-4 exhibited a wide spectrum of antifungal activity against fungi from different genera.

### Real-Time Quantitative PCR Analysis of Gene Expression in AFs Biosynthesis

**FIGURE 9:**
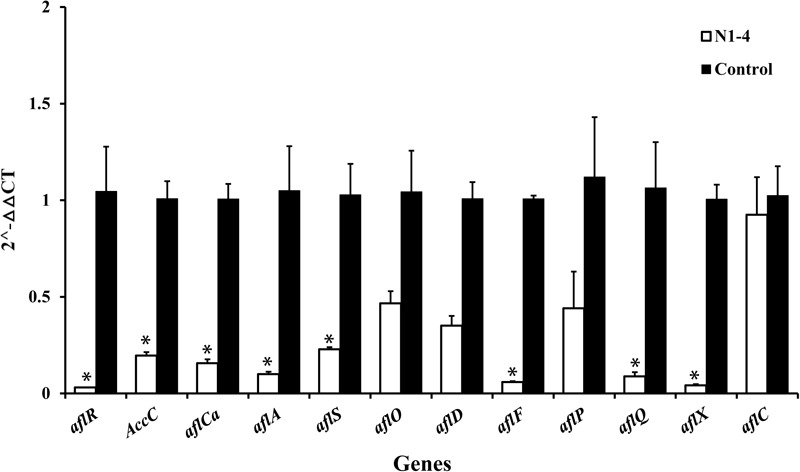
Expression of genes (RT-qPCR) involved in aflatoxin biosynthesis pathway of *A. flavus* effected by volatiles from N1-4. The fold change of genes in N1-4 treatment was calculated with 2^∧^-ΔΔct method. And ΔΔct = ΔCt in N1-4 treatment -ΔCt in control treatment. ^∗^means significant difference at *p* < 0.05 compared to CK treatment.

In RT-qPCR analysis, the expression of 12 genes in N1-4 treatment was all reduced compared to control treatment. Eight genes were significantly down-regulated by 4.39 to 32.25-fold. The results further prove that volatiles from N1-4 greatly repress genes expression in *AFs* biosynthesis (Figure 9), and finally reduce AFs production.

## Discussion

*Aspergillus flavus* can infect many grain, food and feedstuff in storage, and produce toxic and carcinogenic AFs which causes great hazardous to humans and livestock. The control of *A. flavus* and AFs at storage is an urgent and crucial problem to scientists worldwide. However, except for the traditional physical and chemical methods, few effective and safe agents are applied in control *A. flavus* and AFs during storage recently. In our current work, we innovatively prove that *A. faecalis* N1-4 isolated from tea rhizosphere could produce two antifungal volatiles including DMDS and MI, significantly inhibit the mycelia growth and gene expression in aflatoxin biosynthesis, eventually inhibit *A. flavus* infection and AFs production in four grains of high a_w_, and severely damage *A. flavus* cell structure. Thus, we deduce that strain N1-4 and the produced volatiles provide novel and alternative strategies for control *A. flavus* and AFs during storage, and open new views for screening effective biocontrol agents.

*Alcaligenes faecalis* is a gram-negative bacterium, commonly existing in soil and water. These bacteria have been broadly applied in several fields including organic acid production (Yamamoto et al., 1991; He et al., 2009), plant growth promoting (Sayyed and Chincholkar, 2010; Sayyed et al., 2010), NH4^+^-N removal in wastewater (Joo et al., 2005), biodegradation of phenol contamination (Jiang et al., 2007) and biocontrol activity (Shan et al., 2019). For example, Yokoyama et al. (2013) report that *A. faecalis* AD15 produce large amount of hydroxylamine (33.3 mg/L) in synthetic medium, and greatly inhibited the growth of two pathogens including *Pantoea agglomerans* and *Colletotrichum gloeosporioides*. Kakar et al. (2018) report that *A. faecalis* strains Bk1 and P1 greatly suppress the incidence of sheath blight diseases more than 70%. And both of them with phosphate solubilizing activity can produce indoleacetic acid, ammonia, siderophores, enrich the content of mineral nutrients in seedlings and improved plant growth. However, to our knowledge, the application of *A. faecalis* in preventing plant disease at storage has not been reported till now.

As we all know that *A. flavus* as an air-borne fungus produce abundant conidia during infection which spread in a long distance to infect grain and food. Hence, the control of *A. flavus* and AFs in storage is more difficult than other pathogens without conidia. Recently, Ma et al. (2017) report that the biocontrol activity of volatile phase on *A. flavus* is more effective than contact phase. Hence, screening antagonistic volatiles in control post-harvest *A. flavus* and AFs is urgent nowadays. So far, approximately 1,300 microbial volatiles have been obtained from microorganisms and registered in the volatile database^1^ (Lemfack et al., 2014). However, less than 10 volatiles are proved useful in control *A. flavus* or AFs till now. In our current work, two novel antifungal volatiles, DMDS and MI, are identified, and show effective in control *A. flavus in vitro*. They both are first proved effective in control *A. flavus* in grains during storage. Especially for DMDS, it shows the highest relative abundance at 69.90% in N1-4 volatiles, and best antifungal activity to *A. flavus* with MIC at 50 μL/L. MI shows less inhibitory activity compared to DMDS, The MIC for MI is 200 μL/L. These results prove that DMDS with effective antifungal activity will provide more valid and alternative bio-active agents in control *A. flavus* and other fungal pathogens in storage.

Dimethyl disulfide (molecular weight 94 Dalton) is easily spread in storage condition. Some research reported that it is naturally existed in fresh *Allium porrum* and *Romanesco cauliflower* plants (Valette et al., 2003; Sébastien et al., 2004). It is always considered as common and safe compounds, and be used as spice additive in food. Some microbe can also produce DMDS such as *Bacillus cereus* (Huang et al., 2012), *pseudomonas aeruginosa* (Briard et al., 2016), *Serratia odorifera* (Kai and Piechulla, 2010). DMDS with broad resource has been applied in plant disease control field. For example, DMDS shows effective activity in control root knot nematodes and cyst nematodes (Curto et al., 2014; Fritsch et al., 2014; Sasanelli et al., 2014), Recently, Papazlatani et al. (2016) proves that DMDS at low dose (56.4 g m^-2^) could drastically reduce the population of tomato soil borne pathogens *F. oxysporum* and *Rhizoctonia solani*. It also shows great activity to other pathogen including *Sclerotium rolfsii, Verticillium dahliae* and *R. solani* (Fritsch, 2004). Moreover, DMDS can induce systemic resistance in plant, and promote the growth of plant in field (Huang et al., 2012; Meldau et al., 2013; Piechulla et al., 2017). DMDS as novel bio-active compound with antagonistic activity in disease and pest control, plant growth promoting and ISR can be considered as potential biocontrol agent in field and storage.

Additionally, the volatiles produced by strain N1-4 also show broad antifungal activity against other 6 important fungal pathogens including *Fusarium graminearum, F. equiseti, Alternaria alternata, Botrytis cinerea, Aspergillus niger* and *Colletotrichum graminicola*. These fungi belong to different genera of two phylum including ascomycota, deuteromycotina. Each of them can cause great damage to crops, fruits and vegetables in field or storage. Our current work elucidates that volatiles from N1-4 can greatly prevent the growth of these different fungi *in vitro*. It may also inhibit the infection of these fungi in field or storage. Hence, we deduce that strain N1-4 and the produced volatiles may provide novel agents in controlling fungal pathogens and associated mycotoxins in practice.

## Data Availability

GenBank accession numbers for our nucleotide sequences: N1-4: MK972333.

## Author Contributions

A-DG and Y-CL conceived and designed the experiments. A-DG, N-NW, X-WK, and F-YD performed the experiments. A-DG analyzed the data. J-HW, Z-ZY, S-JG, and Y-MZ contributed reagents, materials, and analysis tools. A-DG and M-JH wrote the manuscript.

## Conflict of Interest Statement

The authors declare that the research was conducted in the absence of any commercial or financial relationships that could be construed as a potential conflict of interest.

## Footnotes

1 http://bioinformatics.charite.de/mvoc/
